# Subcritical Water Technology for Enhanced Extraction of Biochemical Compounds from* Chlorella vulgaris*

**DOI:** 10.1155/2016/5816974

**Published:** 2016-06-05

**Authors:** S. A. Awaluddin, Selvakumar Thiruvenkadam, Shamsul Izhar, Yoshida Hiroyuki, Michael K. Danquah, Razif Harun

**Affiliations:** ^1^Department of Chemical and Environmental Engineering, Universiti Putra Malaysia, 43400 Serdang, Malaysia; ^2^Department of Chemical Engineering, Curtin University, 98009 Sarawak, Malaysia

## Abstract

Subcritical water extraction (SWE) technology has been used for the extraction of active compounds from different biomass materials with low process cost, mild operating conditions, short process times, and environmental sustainability. With the limited application of the technology to microalgal biomass, this work investigates parametrically the potential of subcritical water for high-yield extraction of biochemicals such as carbohydrates and proteins from microalgal biomass. The SWE process was optimized using central composite design (CCD) under varying process conditions of temperature (180–374°C), extraction time (1–20 min), biomass particulate size (38–250 *μ*m), and microalgal biomass loading (5–40 wt.%).* Chlorella vulgaris* used in this study shows high volatile matter (83.5 wt.%) and carbon content (47.11 wt.%), giving advantage as a feedstock for biofuel production. The results showed maximum total carbohydrate content and protein yields of 14.2 g/100 g and 31.2 g/100 g, respectively, achieved under the process conditions of 277°C, 5% of microalgal biomass loading, and 5 min extraction time. Statistical analysis revealed that, of all the parameters investigated, temperature is the most critical during SWE of microalgal biomass for protein and carbohydrate production.

## 1. Introduction 

Global energy demands continue to increase at a current annual consumption rate of about 500 Quadrillion Btu (QBtu). Approximately 92% of this consumption demand is met by nonrenewable fossil energy sources which have significant negative impacts on the environment and the economy [1]. Biofuels represent a class of renewable energy with the potential to contribute significantly to the sustainable energy mix required to meet future energy demands. Microalgal biomass is heavily researched as feedstock for the production of different types of biofuels as a result of its fast growth rate, nonedibility, and the capacity to accumulate high concentrations of biochemical compounds such as lipids and carbohydrates for biofuel synthesis [2].

Microalgal primary metabolites, such as proteins, fatty acids, and carbohydrates, are produced intracellularly and entrapped within the cells; thus an effective extraction technology is required to release these biochemical products [3]. The primary metabolites are source of bioactive metabolites, such as vitamins and enzymes, which are commercially beneficial due to their antioxidant, anti-inflammatory, antiangiogenic, antiobesity, and anticancer properties [3]. Commonly used extraction technologies via chemical and mechanical methods include expellers, liquid-liquid extraction (solvent extraction), super-critical fluid extraction (SFE), and ultrasound techniques [4–7].

Expellers are commonly used to extract oil from nuts and seeds [4]. However, they could find applications in the extraction of lipids from dry microalgal biomass. The expeller uses pressure to compress and expel oil from the feedstock biomass. Although this method produces high yields of oil, it is disadvantaged with high energy consumption and prolonged extraction time [4]. Solvent extraction (SE) has demonstrated to be efficient for lipid extraction from microalgal biomass [5]. In this approach, an organic solvent or a mixture of solvents including benzene, cyclohexane, hexane, acetone, and chloroform react with the microalgal biomass [8]. The solvent ruptures and/or penetrates the microalgal cell wall and extracts lipid from the intracellular aqueous medium since lipids have higher solubilities in organic solvents than water. Solvent extract is then subjected to distillation to separate the lipid from the solvent. The solvent can be recycled for further use. Supercritical fluid extraction (SFE) makes use of high pressures to rupture the biomass cells. This method of extraction has proven to be extremely time efficient and has been employed for a wide range of biomass materials [6]. It has been reported that the operating temperature and pressure of SFE do not significantly affect product yield but rather the extraction rate [9, 10]. Andrich et al. [11] compared the polyunsaturated fatty acids (PUFA) extraction yields of SFE and SE using* Spirulina platensis* and reported that SFE demonstrated a higher PUFA yield and fatty acid composition compared to SE. Another promising technology for lipid extraction from microalgal biomass is via ultrasound. This method exposes the microalgal biomass to high intensity ultrasonic waves to create tiny cavitation bubbles around the cells. The collapse of the bubbles emits shockwaves which shatter the cell wall to enable the release of intracellular materials. Wiltshire et al. [7] reported ultrasound extraction yields of 90% for fatty acids and pigments from* Scenedesmus obliquus*. Although ultrasound extraction of lipids from microalgae is quite effective at small scale, numerous technoeconomic challenges arise with large scale applications. This has dwindled interests in the use of ultrasound extraction technology for microalgal biomass extraction outside laboratory environment. One of the recent advancements in the extraction of intracellularly entrapped compounds from biomass materials is the use of subcritical water extraction (SWE) technology. The technology has been demonstrated to offer lower production costs, mild operating conditions, and shorter production duration compared to the aforementioned conventional methods [12–14]. In this technology water reacted as a solvent and catalyst to convert biomass into value added products [15]. Water ionization and dielectric potential are the two characteristics of water which are of unique importance during SWE. As the temperature of water increases, its hydrogen bonding cleaves with decreasing dielectric constant and polarity [16]. This results in an increase in the concentration of hydrogen ion [17]. Extensive studies led by Yoshida and coworkers found that valuable and useful substances such as organic acids, amino acids, proteins, fatty acids, and oils are recoverable through the use of SWE, and the technology can be applied in wastewater treatment. For example, fish waste was liquefied by hydrolysis using subcritical water technology to enable the recovery of organic acids and amino acids and the extraction of fatty acids [18]. Similar results were also obtained with squid waste where free fatty acids containing EPA and DHA were produced during hydrolysis with subcritical water technology [19].

With increasing research interests in microalgal biotechnology through the development of value added products, along with the limited application of SWE on microalgal biomass, this work seeks to investigate the application of SWE for optimal extraction of biochemical compounds from* Chlorella vulgaris*.

## 2. Materials and Methodology

### 2.1. Microalgal Species and Biomass Development

*C. vulgaris* (green microalgae) biomass was used for the extraction process. The microalgal species was obtained from Pure Bulk Inc. (USA) and delivered in a green powdered form with an average particulate size of 100 *μ*m. The powdered microalgal cells were stored in a desiccator until further use.

### 2.2. Characterization of Microalgal Biomass

#### 2.2.1. Proximate Analysis

The moisture, volatile matter, fixed carbon, and ash contents of* C. vulgaris* biomass were determined using a thermogravimetric analyzer (TGA) (TGA/SDTA851, Mettler Toledo, USA). 20 mg of fine biomass powder was placed in alumina crucible and heated inside a furnace. The sample was continuously heated under different conditions of temperature (0–1000°C) and heating rate (5, 10, and 20°C min^−1^) and at a constant gaseous nitrogen (N_2_)/air atmosphere flowing at 25 mL/min.

#### 2.2.2. Ultimate Analysis

The elemental composition of* C. vulgaris* biomass was determined using CHNS analyzer (LECO True Spec CHNS628, USA). Approximately 1.0 mg of dry biomass sample was weighed into a tin capsule and transferred to the autosampler. The temperature was set at 1000°C, and oxygen, nitrogen, and helium were used as the carrier gases.

#### 2.2.3. Scanning Electron Microscopy (SEM)

Scanning Electron Microscopy (SEM) analysis of both intact and extracted biomass fractions was performed using a Hitachi S-3400N Tabletop Microscope configured with an energy dispersive system (EDS) and operated at a voltage of 20 kV. The samples were sputter coated with gold at 5 mA for 45 s prior to SEM analysis. The images were examined under different resolutions ranging from 300 SE to 6.00 kSE.

### 2.3. Subcritical Water Extraction (SWE)

**Figure 1:**
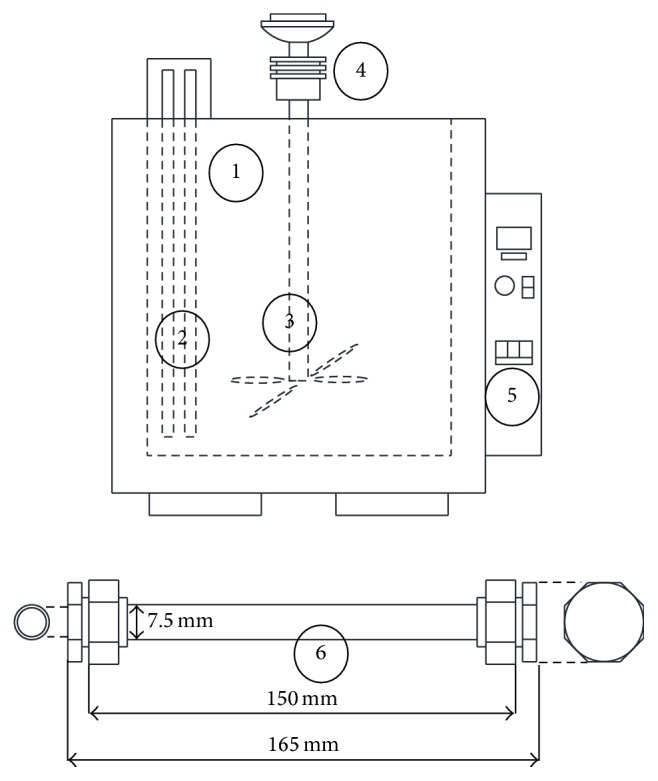
Schematic diagram of the SWE experimental setup. The key components of the system are (1) inner salt bath, (2) heater, (3) stirrer, (4) stirring motor, (5) operation panel, and (6) reactor.

*C. vulgaris* used for SWE was first pulverized into different particle sizes ranging from 38 *μ*m to 250 *μ*m. The different particle size fractions were separated by passing the pulverized sample through a series of cascaded stainless steel sieves. Four process parameters were investigated at five different levels to understand the impacts on microalgal SWE. The four process parameters are temperature (180–374°C), extraction time (1–20 min), particle size (38–250 *μ*m), and biomass loading (5–40 wt.%). The schematic diagram of the SWE system is shown in Figure 1. The dried microalgal biomass at a specific loading concentration was mixed with 6 mL of Milli-Q water and loaded into a stainless steel reaction tube (SUS316) having an inner diameter of 7.5 × 10^−3^ m and 1.5 × 10^−1^ m length. Argon gas was used to purge the reactor for 5 min to release trapped air from the reactor. The reactor tube was then closed tightly with Swagelok caps. The sample filled tube was immersed in a salt bath at a specific temperature for a specific time. After the reaction process, the reactor tube was quenched in a cooling water basin to terminate the reaction.  Table 1 summarizes the process conditions.

### 2.4. SWE Product Analysis

#### 2.4.1. Product Separation

**Figure 2:**
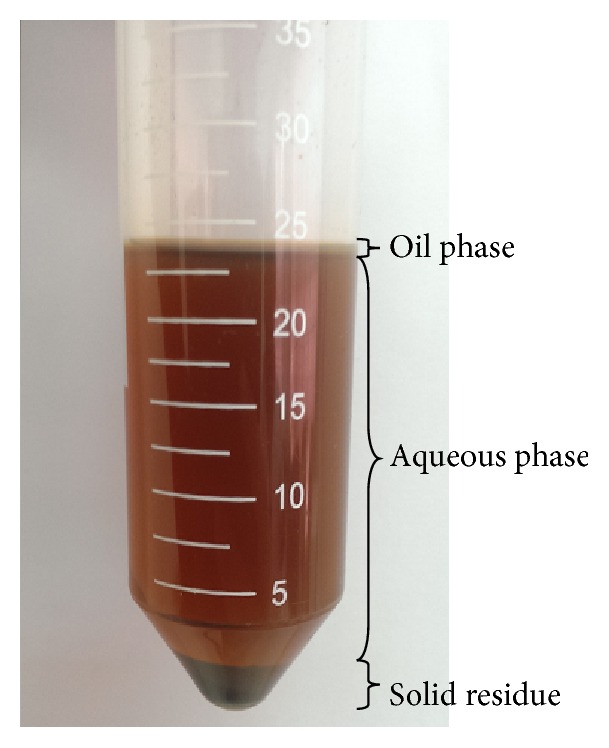
Product fractions from SWE of* C. vulgaris* biomass.

Product extracts from the SWE process were centrifuged (KUBOTA 2420, Japan) at 4000 rpm for 10 min. The centrifuged samples formed three different layers of oil, water, and solid residue as shown in Figure 2. Afterwards, 1.5 mL of hexane was added to the extracted samples for oil separation. The mixture was left for 10 min before recovery by decantation. The process was repeated 8 times for complete oil separation. The residual mixtures were further centrifuged at 4000 rpm for 10 min. The supernatant and solid residue were separated by filtration and stored at −20°C until further analysis [23].

#### 2.4.2. Protein Analysis

Protein concentration was determined using the Lowry method [21]. A serial dilution of Bovine Serum Albumin (BSA) solution was prepared from its stock solution (1 mg/mL). 0.2 mL of BSA solutions with concentrations ranging from 0.05 mg/mL to 1 mg/mL was pipetted into multiple test tubes. 2 mL of alkaline copper sulphate reagent was added to each aliquot and incubated for 10 min. The alkaline copper sulphate reagent consists of Na_2_CO_3_, NaOH, CuSO_4_·5H_2_O, and C_4_H_4_KNaO_6_·4H_2_O. Also, 0.2 mL of Folin Ciocalteu reagent was added to the solution, and the optical density was measured at 660 nm using UV spectrophotometer (UV-160A, SHIMADZU, Japan). Water phase sample was pipetted into test tube to determine protein concentration using this method.

#### 2.4.3. Total Carbohydrate Content

Phenol-sulphuric acid method was used to determine total carbohydrate content [22]. A serial dilution of sugar standard (20–100 mg/L) was prepared from glucose. UV spectrophotometer was used to determine the optical density at 490 nm. 1 mL of sample was mixed with 1 mL of 5% phenol and 5 mL of H_2_SO_4_. The sample was left at ambient conditions for 10 min, and the glass lid covered for 25 min incubation before UV spectrophotometric analysis.

#### 2.4.4. Total Organic Carbon (TOC) Analysis

The TOC of the liquid phase extract sample was analyzed using total organic carbon analyzer (TOC-V CPH, Shimadzu, Japan) [23], where 25 *μ*L of aqueous sample was injected. Hydrogen phthalate of 250 ppm was used as a standard solution for this analysis. 250 ppm of sodium bicarbonate and 250 ppm of sodium carbonate were mixed with the sample aqueous phase and injected into the analyzer. The TOC of the sample was obtained by subtracting the measured inorganic carbon (IC) from the measured total carbon (TC).

### 2.5. Statistical Analysis

Central Composite Design (CCD) was employed to optimize process parameters during SWE in order to maximize product yield. The parameters optimized in this study are temperature, extraction time, particle size of microalgae, and microalgae loading concentration. The results from the experimental work were statistically analyzed using the Statistical Software (Statsoft, v.5.0). The dependent variable for the statistical analysis is the yield of extracted product, whilst the independent variables are the process parameters. The effect of each parameter was analyzed in order to evaluate the synergistic effects of multiple process parameters on maximizing the yield of bioactive compounds.

## 3. Results and Discussion 

### 3.1. Biomass Characterization

#### 3.1.1. Proximate and Ultimate Analysis

Proximate analysis gives a quantitative estimation of the microalgal biomass material composition. The composition of* C. vulgaris* biomass used in this study is divided into four main groups as shown in Table 2. The high volatile matter percentage of microalgal biomass is advantageous for biofuel production [24].

Ultimate analysis generates biomass compositions such as carbon, hydrogen, oxygen, nitrogen, sulphur, and ash through combustion and can be used to investigate the effect of reaction temperature on CHNS ratio (number of atoms to carbon) of the solid residual. During the process, carbon is converted to carbon dioxide; hydrogen to water; nitrogen to nitrogen gas; and sulphur to sulphur dioxide [24]. Table 2 shows the CHNS percentage of SWE treated and raw samples of the biomass. The SWE treated samples were exposed to SWE under the conditions of 277°C temperature, 5 min extraction time, 90 *μ*m biomass particulate size, and 5% biomass loading concentration. The carbon content increased by 25% with SWE treatment compared to the intact cells, indicating that the extraction process resulted in some degree of biomass disruption and this enabled a higher amount of carbon to be available for combustion. The results of ultimate compositions of* C. vulgaris* are also compared with other biomass (Table 3). The* C. vulgaris* biomass provides higher carbon percentage, hence indicating that it contains substantial amounts of lipid, cellulose, and hemicellulose which are good for biodiesel and bioethanol production [25].

#### 3.1.2. Scanning Electron Microscopy (SEM)

**Figure 3:**
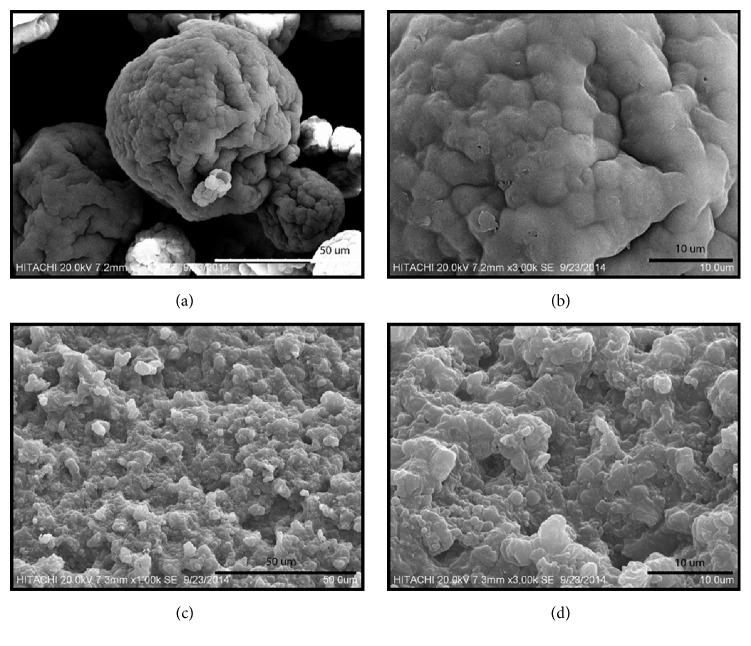
SEM images of nontreated (a and b) and SWE treated (c and d)* C. vulgaris* biomass samples. The images were captured at the same voltage of 20 kV and multiple resolutions of 7.2 mm × 1.00 kSE (a), 7.2 mm × 3.00 kSE (b), 7.3 mm × 1.00 kSE (c), and 7.3 mm × 3.00 kSE (d). SWE treatment occurred at 215°C for 6 min using 28 wt.% microalgal biomass with particulate size of 180 *μ*m.

Figure 3 shows the surface morphology of SWE treated and nontreated* C. vulgaris* biomass samples. From the images, it can be deduced that the biomass cells of the nontreated sample appeared intact and agglomerated, forming a large spherical cell mass. The diameter of the smallest cell measured ~5 *μ*m, whereas the biggest cell measured ~10 *μ*m. After SWE treatment, the biomass cells looked ruptured and segregated into individual particulate cells. The strength of hydrogen bonding existing in water decreases with increasing temperature, and this decreases its dielectric constant [26]. This further decreases water polarity, allowing nonpolar compounds to dissolve in water [12, 27]. Water ionization rises as water temperature increases and this increases the concentration of hydrogen ion which enables water to act as an acid catalyst. Thus, with the water temperature increase, nonpolar compounds in* C. vulgaris* biomass were dissolved in water, resulting in the segregation and disruption of the biomass cells.

### 3.2. SWE Product Analysis

#### 3.2.1. Protein

**Figure 4:**
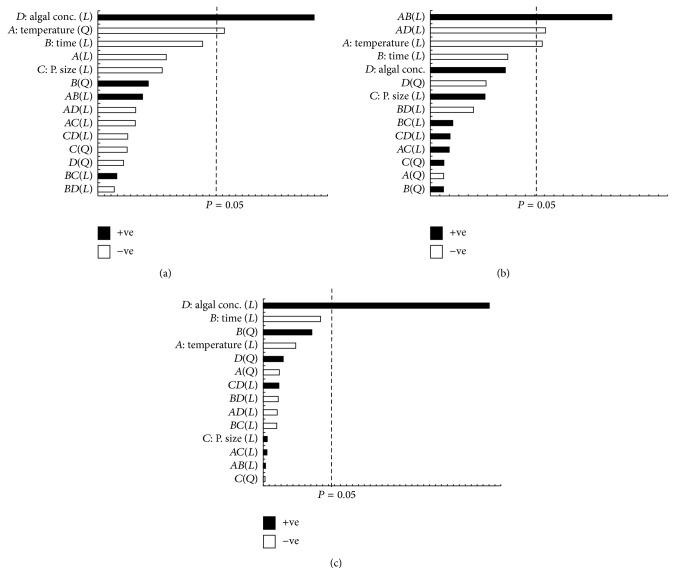
Standard Pareto chart of the process parameter for bioactive compound extraction from* C. vulgaris*, (a) protein, (b) total carbohydrate content, and (c) TOC. +ve and −ve sign describe the effect of the selected parameter that gives a positive and negative impact on the bioactive compounds extraction.

The highest protein concentration of 31.16 g/100 g was obtained when 5 wt.% microalgal biomass with 90 *μ*m particulate size was treated under 277°C temperature condition for 5 min. The statistical analysis indicated that the optimum conditions to maximize protein yield from SWE of* C. vulgaris* are temperature (281°C), extraction time (17 min), particle size (75 *μ*m), and microalgae loading (45 wt.%).  Table 4 shows the protein yield characteristic equation generated for SWE of the microalgal biomass as a function of the process parameters. It was determined that the most significant process parameters affecting protein extraction from the biomass were microalgal loading concentration. The microalgal loading concentration demonstrated a positive effect whilst the other process parameters demonstrated a negative effect as shown in Figure 4(a). Increasing the microalgal loading concentration in SWE increases protein yield as high biomass loading concentrations present an increased amount of available proteins for extractions. Proteins denature at high temperatures, and this explains the negative effect of decreasing protein yield under high SWE temperature conditions. Protein denaturation happens when the supplied heat increases the molecular kinetic energy of the protein, causing it to vibrate rapidly and destroying the protein tertiary structure through the cleavage of its hydrogen bonds and nonpolar hydrophobic interaction [28].  Figure 6(a) shows the relationship between the independent process variables and the protein yield. The yield of protein increases with increasing microalgal loading concentration. Increasing the extraction temperature to the optimum point (280–300°C) results in increasing protein yield. However, a further increase in temperature results in decreasing protein yields, and this is due to thermal denaturation as explained earlier.

#### 3.2.2. Carbohydrate

**Figure 5:**
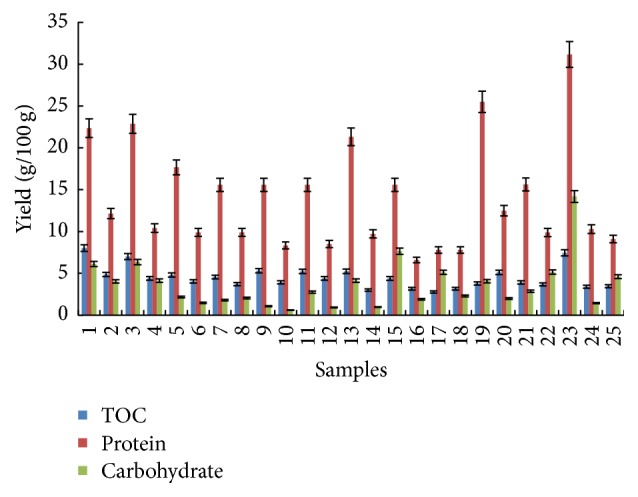
Total carbohydrate, protein, and TOC yields from different SWE treatment conditions of* C. vulgaris* biomass.

**Figure 6:**
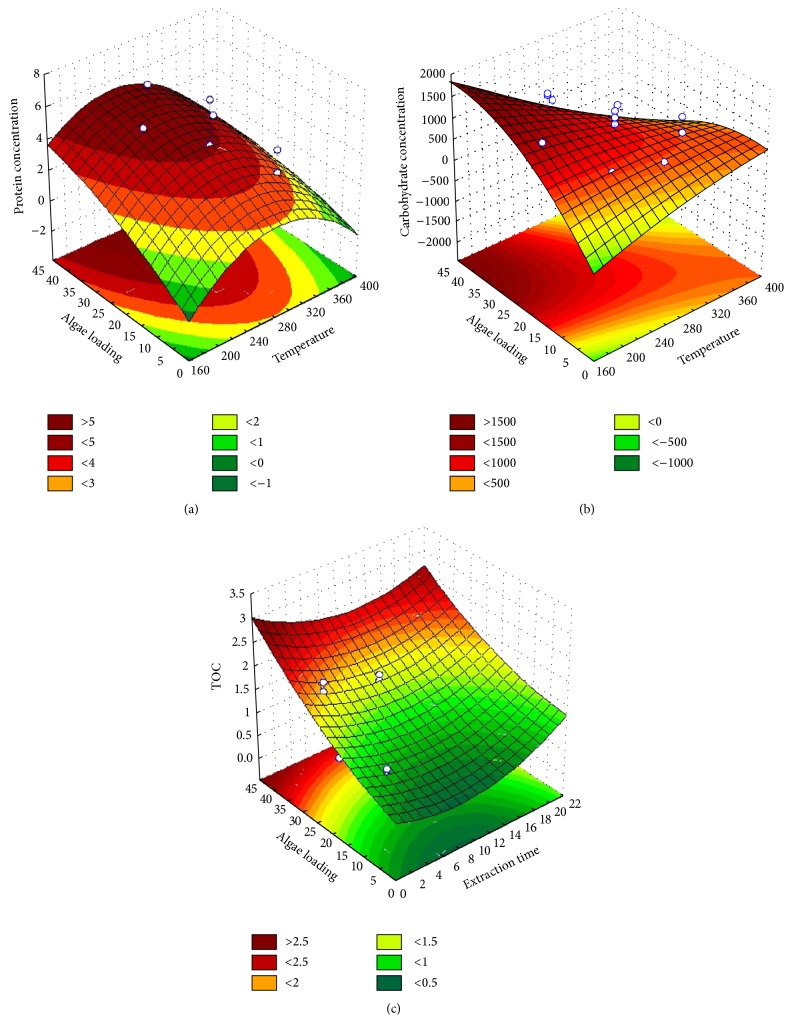
Fitted response surface curves for (a) protein, (b) total carbohydrate, and (c) TOC.

Figure 5 shows a summary of the total carbohydrate yields obtained from the experimental design. The maximum carbohydrate yield of 14.18 g/100 g was achieved under SWE conditions of 277°C for 5 min with 5 wt.% microalgal biomass having 90 *μ*m particulate size. The lowest carbohydrate yield of 0.255 mg/mL was obtained at 326°C for 3 min with 30 wt.% microalgae biomass with particulate size 75 *μ*m. The microalgae biomass carbohydrate has previously been reported to have different types of polysaccharides, including cellulose, xylose, galactose, and arabinose, entrapped in the cell walls [29]. Based on the statistical analysis in Figure 4(b), the carbohydrate yield was influenced by extraction temperature, time, and microalgal biomass loading. The extraction temperature and microalgal biomass loading demonstrated a negative effect on carbohydrate yield whilst the extraction time demonstrated a positive effect. In brief, low temperature, longer extraction time, and low biomass loading during SWE support high carbohydrate yields. Extraction of carbohydrate gives negative effects because carbohydrate rapidly decomposed when exposed to higher temperatures [30, 31]. Also, biomass loading has an effect on carbohydrate extraction as the yield decreases when the sample is concentrated. The sample was not extracted well when the amount of sample inside the reactor is too concentrated. Microalgae have rigid cell walls which are hard to break; in order to increase the efficiency of carbohydrate extraction, longer extraction time is needed [32, 33]. According to the statistical analysis, the optimum SWE conditions for maximum carbohydrate extraction yield were 302°C temperature, 8 min extraction time, 38 *μ*m particulate size, and 16 wt.% microalgal biomass loading. The equation for the optimum conditions of carbohydrate extraction yield is shown in Table 5. The relationship between the microalgal biomass loading and the extraction temperature is shown in Figure 6(b). Based on the surface plot, it can be seen that the maximum carbohydrate yield was achieved when the concentration of biomass loading and extraction temperature were higher. The highest carbohydrate concentration of ~2000 mg/L at 40–45% microalgal biomass loading was obtained under the temperature range of 100 to 200°C.

#### 3.2.3. Total Organic Carbon (TOC)

Total organic carbon (TOC) analysis determines the concentration of total organic carbon covalently bonded in the microalgal biomass. Amongst all the process parameters investigated, TOC of the biomass was only influenced by the loading concentration of the microalgal biomass as shown in Figure 4(c). With increasing concentration of microalgal biomass loading, the quantity of organic carbon under covalent bonding within the biomass increases. This can be seen from the surface plot in Figure 6(c). From the experimental results, the highest concentration of TOC of 8.01 g/100 g was obtained for the sample exposed to SWE treatment 229°C for 3 min with 10 wt.% microalgal biomass having 75 *μ*m particle size. Figure 5 presents a summary of TOC yield. The characteristic equation relating the optimum conditions of TOC yield is presented in Table 5.

### 3.3. ANOVA

Analysis of variance (ANOVA) for the responses is presented in Table 5. *R* ^2^ values for the selected responses were 0.87, 0.81, and 0.88 for protein, total carbohydrate content, and TOC, respectively. The observed *R* ^2^ values, which were above 0.75, indicated the aptness of the model. The quadratic models presented in Table 4 are, therefore, considered as a satisfactory interpretation to the experimental data.

## 4. Conclusion 

This work investigated the optimum conditions to extract biochemical compounds, mainly protein and total carbohydrate, from* C. vulgaris* biomass using SWE. From the results obtained, microalgal loading concentration was the most significant parameter affecting protein yield, whereas extraction temperature, biomass loading, and extraction time affected carbohydrate yield. The results obtained demonstrate the potential of SWE of microalgal biomass for large scale production of biochemical compounds, such as proteins and carbohydrates, that have wide applications in production of algae-based biofuels and other useful materials. From economic and environmental perspectives, SWE has proven attractive in recovering valuable biochemical materials from a wide range of biomass feedstocks. The use of SWE for the generation of bioproducts from microalgal biomass is expected to herald future sustainable bioextraction technologies, and this work contributes significantly to the validation of SWE technology for optimal biochemical products extraction.

## Figures and Tables

**Table 1:** CCD for *C. vulgaris *SWE experimental conditions.

Sample	Temperature (°C)	Extraction time (min)	Particle size (*µ*m)	Concentration microalgae (%)
1	229 (−1)	3 (−1)	75 (−1)	10 (−1)
2	229 (−1)	3 (−1)	75 (−1)	30 (+1)
3	229 (−1)	3 (−1)	180 (+1)	10 (−1)
4	229 (−1)	3 (−1)	180 (+1)	30 (+1)
5	229 (−1)	10 (+1)	75 (−1)	10 (−1)
6	229 (−1)	10 (+1)	75 (−1)	30 (+1)
7	229 (−1)	10 (+1)	180 (+1)	10 (−1)
8	229 (−1)	10 (+1)	180 (+1)	30 (+1)
9	326 (+1)	3 (−1)	75 (−1)	10 (−1)
10	326 (+1)	3 (−1)	75 (−1)	30 (+1)
11	326 (+1)	3 (−1)	180 (+1)	10 (−1)
12	326 (+1)	3 (−1)	180 (+1)	30 (+1)
13	326 (+1)	10 (+1)	75 (−1)	10 (−1)
14	326 (+1)	10 (+1)	75 (−1)	30 (+1)
15	326 (+1)	10 (+1)	180 (+1)	10 (−1)
16	326 (+1)	10 (+1)	180 (+1)	30 (+1)
17	180 (−2)	5 (0)	90 (0)	20 (0)
18	374 (+2)	5 (0)	90 (0)	20 (0)
19	277 (0)	1 (−2)	90 (0)	20 (0)
20	277 (0)	20 (+2)	90 (0)	20 (0)
21	277 (0)	5 (0)	38 (−2)	20 (0)
22	277 (0)	5 (0)	250 (+2)	20 (0)
23	277 (0)	5 (0)	90 (0)	5 (−2)
24	277 (0)	5 (0)	90 (0)	40 (+2)
25	277 (0)	5 (0)	90 (0)	20 (0)

**Table 2:** Proximate analysis and ultimate analysis of *C. vulgaris *biomass.

Proximate analysis	Composition (wt.%)	
Moisture	7.60 ± 0.1	
Volatiles	83.50 ± 0.3	
Fixed carbon	3.80 ± 0.1	
Ash	6.60 ± 0.2	

Ultimate analysis	Composition (wt.%)	
	Raw sample	SWE treated sample

Carbon (C)	47.11 ± 0.5	58.88 ± 0.6
Hydrogen (H)	7.47 ± 0.3	8.55 ± 0.2
Oxygen (O)	37.16 ± 0.3	25.03 ± 0.5
Nitrogen (N)	8.26 ± 0.4	7.54 ± 0.1
Sulphur (S)	—	—
Ash	—	—

**Table 3:** Ultimate composition of biomass samples.

Biomass	Ultimate analysis (wt.%)					Reference
	C	H	N	S	O	
*C. vulgaris*	47.11 ± 0.5	7.47 ± 0.3	8.26 ± 0.4	—	37.16 ± 0.3	This study
Rice bran	44.40	7.11	2.16	—	46.30	[20]
Jatropha shell	42.45	5.12	1.60	—	50.83	[21]
*Sargassum *sp.	26.70	4.23	1.35	0.19	67.53	[22]

**Table 4:** Generated characteristic equations for optimum product yields from SWE treatment of *C. vulgaris* biomass.

Extraction product	Formula of optimum product^*∗*^
Protein	5.707608*d* − 1.87442*a* ^2^ − 1.35416*b* − 1.89065*a* − 01.96542*c* + 0.8136935*b* ^2^ + 1.22666*ab* − 1.05*ad* − 1.04515*ac* − 0.623285*cd* + 0.4767914*c* ^2^ + 0.9863567*d* ^2^ − 0.335209*bd* − 0.302402*bc*

Total carbohydrates	3.822194*ab* − 2.4369*ad* − 2.35744*a* − 1.63113*b* + 1.579538*d* − 1.1742*d* ^2^ + 1.153092*c* − 0.908555*bd* + 0.4734103*bc* + 0.4169475*cd* + 0.3989977*ac* + 0.2869922*c* ^2^ − 0.280178*a* ^2^ + 0.2793208*b* ^2^

TOC	7.607638*d* − 1.92788*b* + 1.635175*b* ^2^ − 1.09245*a* + 0.672907*d* ^2^ − 0.53976*a* ^2^ + 0.5287401*cd* − 0.50333*bd* − 0.471897*ad* − 0.453396*bc* + 0.1342441*c* + 0.1244762*ac* + 0.0741781*ab* − 0.056626*c* ^2^

^*∗*^ *a*: extraction temperature; *b*: extraction time; *c*: particle size; *d*: microalgal biomass loading.

**Table 5:** Analysis of variance (ANOVA) for the different components.

Response variables^*∗*^	Protein (%)			Total carbohydrate (%)			TOC (%)		
	SS	DF	*P*	SS	DF	*P*	SS	DF	*P*
*Linear*									
*a*	1.22908	1	0.0879	527185	1	0.0401	73767	1	0.3003
*b*	0.63051	1	0.2055	252382	1	0.1340	229731	1	0.0827
*c*	1.32821	1	0.0777	126127	1	0.2757	1114	1	0.8959
*d*	11.2011	1	0.0002	236669	1	0.1453	3577326	1	0.00002
*Square*									
*a* ^2^	1.20806	1	0.0903	7446	1	0.7851	18008	1	0.6012
*b* ^2^	0.22765	1	0.4348	7401	1	0.7857	165268	1	0.1331
*c* ^2^	0.07816	1	0.6438	7813	1	0.7800	198	1	0.9560
*d* ^2^	0.33452	1	0.3472	130787	1	0.2675	27970	1	0.5164
*2-way interaction*									
*ab*	0.51737	1	0.2480	1385817	1	0.0034	340	1	0.9423
*ac*	0.37559	1	0.3205	15102	1	0.6982	958	1	0.9034
*ad*	0.37908	1	0.3184	557228	1	0.0358	13764	1	0.6471
*bc*	0.03144	1	0.7685	21259	1	0.6461	12706	1	0.6600
*bd*	0.03864	1	0.7444	78304	1	0.3845	15659	1	0.6256
*cd*	0.13358	1	0.5470	16491	1	0.6855	17280	1	0.6085
Lack of fit	3.4338	10		948594	10		618100	10	

Total	26.3276	24		5074315	24		5320338	24	
*R* ^2^	0.8694			0.81306			0.8838		
Adj-*R* ^2^	0.68656			0.55134			0.72118		

^*∗*^ *a*: extraction temperature; *b*: extraction time; *c*: particle size; *d*: microalgal biomass loading; SS: sum of squares; DF: degrees of freedom; *P*: probability.
